# Hypernatremia secondary to soluble paracetamol use in an elderly man: a case report

**DOI:** 10.4076/1757-1626-2-6707

**Published:** 2009-06-29

**Authors:** Keith Siau, Arun Khanna

**Affiliations:** Department of Medicine, Musgrove Park HospitalTaunton, TA1 5DAUK

## Abstract

**Introduction:**

Soluble (effervescent) paracetamol is routinely given to elderly patients for convenience. A daily dose of 4 gm in this soluble formulation can contain up to 8.7 gm of sodium chloride, which exceeds the threshold recommended by the World Health Organization. Hypernatremia secondary to soluble paracetamol has rarely been reported. We describe an elderly patient who developed hypernatremia shortly after taking soluble paracetamol.

**Case presentation:**

A confused 89-year-old man with back pain secondary to metastatic prostatic carcinoma was prescribed soluble paracetamol. Ten days later, his serum sodium concentration had increased from 142 mmol/L to 165 mmol/L. Soluble paracetamol was withdrawn shortly before he died, and was believed to have contributed to his hypernatremia.

**Conclusion:**

Hypernatremia is associated with high morbidity and mortality. Clinicians should be aware of the high sodium chloride content in soluble paracetamol, which can precipitate hypernatremia in elderly patients with impaired renal function.

## Introduction

Soluble (effervescent) paracetamol is routinely given to elderly patients. A daily dose of 4 gm in this soluble formulation can contain up to 8.7 gm of sodium chloride, which exceeds the recommendations from the World Health Organization (WHO). Hypernatremia secondary to soluble paracetamol is rare. We describe an elderly patient who developed hypernatremia shortly after taking soluble paracetamol.

## Case presentation

An 89-year-old Caucasian male, who was taking regular paracetamol for chronic lower back pain, developed new ailment. He was found to have prostatic carcinoma with spinal metastases, and was given cyproterone acetate and goserelin. His past medical history was significant only for mild congestive heart failure. He was not taking other medications. His serum sodium concentration was 142 mmol/L, urea 8.8 mmol/L, and serum creatinine 130 μmol/L. His creatinine clearance (Cockcroft-Gault method) was 33 ml/min/70 kg and his estimated glomerular filtration rate (eGFR) was 48 ml/min/1.73 m^2^.

His oral intake had deteriorated on day 11. He was given soluble paracetamol 1 gm four times daily for convenience. Despite initial supplementation with maintenance intravenous fluids (0.9% saline and 5% dextrose), a subcutaneous infusion had to be provided, as intravenous access became more difficult. By day 21, the serum sodium concentration was 165 mmol/L, potassium 3.4 mmol/L, urea 8.4 mmol/L, and creatinine 120 μmol/L were noted. He had been normotensive, with positive fluid balance and good urine output throughout his admission. When hypernatremia was noted, dehydration was immediately suspected to be the cause, and aggressive fluid resuscitation was started. However, this failed to correct the hypernatremia. After an online literature search, soluble paracetamol was thought to be culpable. A suspension formulation was substituted and the paracetamol was withdrawn over the next 3 days.

His serum sodium concentration stabilised at 161, 159, and 160 mmol/L over these 3 days. By day 24, the paracetamol had been stopped. He suffered severe clinical deterioration through sepsis of unknown origin and worsening renal function (urea 13.2 mmol/L, creatinine 165 μmol/L, eGFR 33 ml/min/1.73 m^2^). He died on day 30.

## Discussion

The temporal relation between soluble paracetamol administration and the development of hypernatremia, in the presence of stable renal function, suggests that our patient may have incurred soluble paracetamol-induced hypernatremia.

Elderly patients constitute a high percentage of the total inpatient population. Renal function in older people usually falls with increasing age, compromising the handling of solutes and electrolytes by the kidneys [1]. Hypernatremia, defined as a serum sodium concentration over 145 mmol/L, is associated with a high mortality in elderly people (42-75%), especially when acquired acutely in hospital [1-3]. Morbidity in survivors is high, with neurological deficits if hypernatremia is severe. It is therefore of paramount importance for health-care professionals to recognise, prevent, and treat the cause of hypernatremia early.

High sodium intake has been an especial cause of concern in the elderly, as chronic excess sodium intake has been positively linked with hypertension and associated co-morbidities. Current WHO guidelines stipulate that the daily intake of sodium should not exceed 2 g, which is equivalent to 5 gm of sodium chloride [4]. Considering that a tablet of soluble paracetamol (Panadol) contains 18.6 mmol (427 mg) of sodium [5], 8 tablets of soluble paracetamol a day will equate to 148.8 mmol (3.4 gm) of sodium or 8.7 gm of salt, which exceeds WHO guidelines. Soluble co-codamol (manufactured by Neolab) contains 19.1 mmol (438 mg) of sodium per tablet [5], which corresponds to a maximum of 8.9 gm of sodium chloride per day. Several frequently used over the counter medications, such as antacids and laxatives, also contain generous amounts of sodium. For example, Alka Seltzer Effervescent Antacid tablets contain 19.4 mmol (445 mg) of sodium per tablet (maximum of 8 a day). Movicol, a commonly used laxative, contains 8.1 mmol (187 mg) of sodium per sachet [5]. A list of high sodium preparations has been compiled by UK medicines information pharmacists and is available on the National Electronic Library for Medicines website [5].

Various cases of hypernatremia due to excessive oral sodium consumption have been described. These include cases due to co-codamol [6], sodium phosphate [7], and soluble paracetamol [8]. There have also been reports of hypertension in patients taking long-term soluble paracetamol [9]. Even after accounting for underreporting of hypernatremia secondary to excessive sodium consumption, such cases are rare. However, in all these cases hypernatremia developed in patients with renal insufficiency, and several have been described in children. This suggests that there are homoeostatic mechanisms for maintaining serum sodium, which can be overcome when renal handling of solute load is compromised. In our patient, poor oral intake was a further contributory factor.

Both health-care professionals and patients should be aware of this pitfall. Care should be taken when prescribing for patients in whom high sodium consumption is contraindicated, especially those with hypertension, heart failure, hypernatremia, or renal insufficiency. Moreover, drug manufacturers should clearly communicate salt concentrations to all parties concerned.

## Abbreviations

eGFR: Estimated glomerular filtration rate
WHO: World Health Organization
